# Cockroaches Probably Cleaned Up after Dinosaurs

**DOI:** 10.1371/journal.pone.0080560

**Published:** 2013-12-04

**Authors:** Peter Vršanský, Thomas van de Kamp, Dany Azar, Alexander Prokin, L'ubomír Vidlička, Patrik Vagovič

**Affiliations:** 1 Geological Institute, Slovak Academy of Sciences, Bratislava, Slovakia; 2 Arthropoda Laboratory, Paleontological Institute, Russian Academy of Sciences, Moscow, Russia; 3 ANKA/Institute for Photon Science and Synchrotron Radiation (IPS), Karlsruhe Institute of Technology (KIT), Eggenstein-Leopoldshafen, Germany; 4 Faculty of Science II, Natural Sciences Department, Lebanese University, Fanar, Fanar-Matn, Lebanon; 5 I.D. Papanin Institute for biology of inland waters Russian Academy of Sciences, Borok, Russia; 6 Voronezh State University, Voronezh, Russia; 7 Institute of Zoology, Slovak Academy of Sciences, Bratislava, Slovakia; 8 Department of Biology, Faculty of Education, Comenius University, Bratislava, Slovakia; 9 Institute of Multidisciplinary Research for Advanced Materials, Tohoku University, Japan; State Natural History Museum, Germany

## Abstract

Dinosaurs undoubtedly produced huge quantities of excrements. But who cleaned up after them? Dung beetles and flies with rapid development were rare during most of the Mesozoic. Candidates for these duties are extinct cockroaches (Blattulidae), whose temporal range is associated with herbivorous dinosaurs. An opportunity to test this hypothesis arises from coprolites to some extent extruded from an immature cockroach preserved in the amber of Lebanon, studied using synchrotron X-ray microtomography. 1.06% of their volume is filled by particles of wood with smooth edges, in which size distribution directly supports their external pre-digestion. Because fungal pre-processing can be excluded based on the presence of large particles (combined with small total amount of wood) and absence of damages on wood, the likely source of wood are herbivore feces. Smaller particles were broken down biochemically in the cockroach hind gut, which indicates that the recent lignin-decomposing termite and cockroach endosymbionts might have been transferred to the cockroach gut upon feeding on dinosaur feces.

## Introduction

The Triassic, Jurassic and Early Cretaceous terrestrial ecosystems differed from extant ecosystems for various reasons, one of them being the presence of gigantic reptiles. The energy flow was principally less efficient (more rapid) and also the general appearance of the landscape was dissimilar [1], [2]. Grasses, flowers with their fruits, large butterflies, and before the latest Jurassic, all eusocial insects (cockroaches, termites, ants, bees) were absent [3], [4]. Discerning between dinosaur feces decomposers (which were not identified until now) is also essential as it changes the general appearance of our assemblage reconstructions. Moreover, the problem is of a principal, systemic importance. If nothing fulfilled this role, a large amount of dung would prevent soil regeneration just as it suffocated the pasture systems and prevented grass regeneration in present-day Australia [5]. Grasses were absent before the Early Cretaceous, but such influence will definitely alter extinct cenoses similar to some extent to the variety of living fern groups or perhaps taxa such as *Gnetum* and *Ephedra*. On the other hand, bird droppings are known to significantly (often positively) influence vegetation composition of ombrotrophic bogs [6]. Late Cretaceous biomes actually contain grasses and silicified plant tissues (phytoliths) preserved in the Maastrichtian coprolites (presumably from titanosaurid dinosaurs) from the Lameta Formation in India show that at least five taxa from extant grass (Poaceae) subclades were present during the latest Cretaceous [7].

Was the Mesozoic world full of sterile dinosaur dung, clean as a modern forest, or transitional between these two extremes? Circumstantial evidence of dinosaur (probably hadrosaur) coprolites [8], [9] suggests that feces were used. The absence of dung-beetles during the Triassic and near-absence during most of the Jurassic [10] (roughly half of the age of dinosaurs) and their radiation associated only with the spread of modern grasslands [1] is still under discussion [2].

Feces have a greater capacity to retain moisture than the parent plant tissue [11] and coprophages exploit the microbial consortia concentrated on these recycled cellulose-based foodstuffs; the microorganisms serve not only as a source of nutrients and gut mutualists, but they also pre-digest recalcitrant substrates [12]. Microbial dominance is so pronounced that fecal pellets may be considered as living organisms [12]. They consist largely of living cells, they consume and release nutrients and organic matter, and they serve as food for animals higher on the food chain [13].

Any excrement is a valuable source of nitrogen, and its amount must have been huge [14] at least seasonally [15], during the age of dinosaurs. Each single separate dung might have had a volume of 7 liters [8]. Probably an important feature of dinosaur and pterosaur excrements (as in birds and reptiles when compared with mammals) was the large proportion of nitrogen compared with phosphorus [16]. The association with urine and thus with a high concentration of phosphoric acid, oxalic and carbonic acids and salts, primarily sodium chloride, leads to the recent conclusion about the association of dung-beetles and coprophagy with mammals (not with dinosaurs) since the very beginning [17]. On the other hand, some common (11 of the 15 deposits) fossilised dinosaur coprolites contain 13–85% of rotting conifer wood with only 0.20–0.30% of nitrogen (conifers are utilized by the living cockroach *Cryptocercus* – the most important wood-decomposing cockroach) with its attendant microbial and detritivore fauna and thus augmented the resource options of Cretaceous ecosystems that lacked fodder provided by grasses and other derived angiosperms [8], [18]. The consistency of the coprolites during the deposition varied from fairly cohesive to viscous liquid and fluid to some extent – those containing a significant amount of wood are most easily recognizable as their high wood content prevented degradation [8].

In addition to dung, it has recently been proposed that the density of sauropods was high enough to produce the amounts of methane necessary for sustaining the warm climate during the Mesozoic [19].

**Figure 1:**
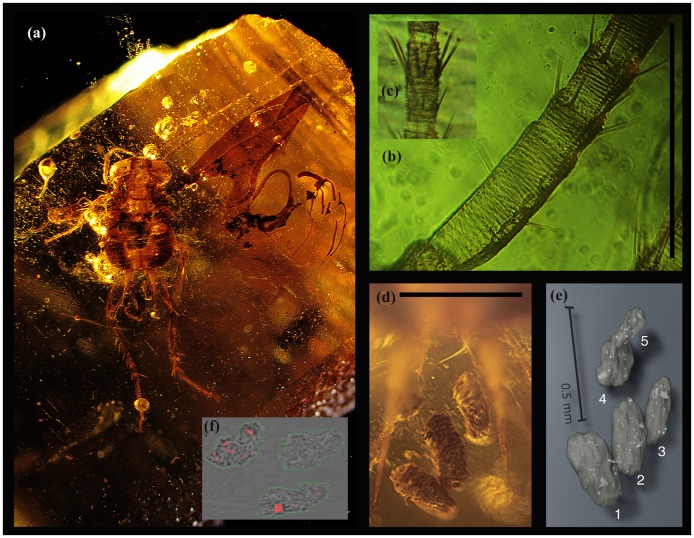
Dinosaur-age cockroach of the extinct family Blattulidae. (A **–** head to leg end length: 3.8 mm) with antennal sensory system (B, C) and five preserved coprolites (D – optical, E – surface rendering of numbered coprolites and dense particles based on the image stack from synchrotron X-ray microtomography; F – ST orthoslice with labelled boundaries and fragments). Lebanon amber 1094A-I. Scales 0,5 mm.

The cockroach family Blattulidae, described by Vishniakova [20] originated in the Late Triassic and constitutes a (co-)dominant group of insects (∼1%) throughout the whole Jurassic and Cretaceous [21]. They are often completely preserved [22]–[24] and contributed to knowledge of some general patterns such as the decreasing variability of species over time, and mass mutations [25], [26]. The Blattulidae constitute the sole cockroach fossils preserved in several Cretaceous localities such as Shin Khudukh and some others in Mongolia and Verchnebureinskaja Vpadina in Russia, and are the dominant insect fossils in diverse Mesozoic ambers [27], [28]. The hypothesis tested and supported in the course of the present research was the heterogeneous character of the diet of these Mesozoic cockroaches (in contrast to homogeneous one of all the studied Cenozoic and present ones). There are numerous Tertiary (Cenozoic) cockroaches preserved with the gut-content, but all of them have a homogeneous diet. The same holds for the studied living cockroaches. The occurrence of any wood (digested twice, a second time by cockroaches, after it was previously digested by herbivores; Figs. 1E, S1) was entirely unexpected.

Protozoan cysts and helminth eggs preserved in the Early Cretaceous *Iguanodon* coprolite represent the only reported case of dinosaur parasites [29], but the discovered trophic relation of dinosaur-age vertebrate herbivore and insects might appear important also due to the structuring of the extinct ecosystems via parasites (and pathogens) transferred. Trophic association of Mesozoic vertebrates and insects suggest endoparasite transfer as well.

A similar transfer is known from numerous living species, e.g., from *Blatta orientalis* and *Periplaneta americana* feeding on human excrement that contained cysts of *Chilomastix mesnili* and rats eating food that had been contaminated with feces from these cockroaches became infected with this protozoan [30].

## Materials and Methods

The material studied herein is from Mdeirij-Hammana, Baabda District Governorate Mount Lebanon, Central Lebanon - detailed coordinates for the localities of completely studied specimens (mostly immatures: (59, 76A, 623i-m, 778AB, 799, 800, 810CD, 845AB, 934AB, 1062, 1274B,D, FAL -3C (Falougha), 133.C, JEZ.F-14 (Wadi Jezzine, Jezzine District, Governorate Southern Lebanon), 1669-B, RIH-33 (Rihane outcrop, Jezzine District, Governorate Southern Lebanon), (deposited at the Lebanese University); AMNH Lebaneese amber 22, 77, 84, 91 (Bcharreh District, Governorate North of Lebanon; Jouar Ess-Souss, Bkassine, Jezzine District, Governorate Southern Lebanon, all deposited in the American Museum of Natural History), *J. lebani* holotype (Jouar Ess-Souss, Bkassine, Jezzine District, Governorate Southern Lebanon, Acra collection) can not be revealed due to site protection [31], in a Lower Cretaceous (ca. 120 Ma) amber-bearing deposit. An enicocephalid assassin bug, three ceratopogonid biting midges, and two male coccids occur as syninclusions. Examined specimen (1094A-I) was not embedded in epoxy resin due to ST examination, but for photography a drop of maple sirup and a coverslip glass was attached to see inside. It is deposited at the Lebanese University, Faculty of Sciences II, Lebanon. We performed a microtomographic scan of the amber piece (0.185 g, well transparent dark yellow-red sample) at the full-field X-ray imaging station TopoTomo beamline of the ANKA light source. The scan covered 180 angular degrees with 2,800 radiographic projections measured. We used a filtered white beam radiation with a spectrum peak at ∼20 keV. A sample-to-detector distance of 35 cm resulted in both absorption contrast and edge enhancing phase contrast in the projection images. These were recorded by an indirect detector system based on a scintillator coupled to an optical microscope and a CCD detector [32]. The magnification factor of the optical microscope was 22.4 which led to an effective pixel size of 0.4 µm with attached CCD camera pco.4000 with 4008×2672 pixels. We processed each radiographic projection using a single distance phase retrieval algorithm [33] integrated in ANKA phase plugin [34] for ImageJ and reconstructed the volume by PyHST reconstruction software [35]. The triangle algorithm is unknown, but the original surfaces contain so many polygons that the details lost to a reduction to 10% are negligible.

For segmentation of the coprolites we used software Amira 5.4. After loading the volume data as an image stack of virtual slices, we labelled the whole coprolites and the dense particles with the segmentation editor of the program. We exported and reassembled the surface models from the labels with the software Cinema 4D R12. Volumes were calculated from the polygon meshes using the GeoTools2010 plug-in.

Before creating the interactive 3D graphics, we reduced the surface polygons once more to 10%. The objects were saved as Collada files and opened with the software Right Hemisphere® Deep Exploration 6. After creating the object hierarchy, we saved the data as Universal 3D files, opened with Adobe® Acrobat® 9 Pro Extended, and integrated into PDF files.

## Results

**Figure 2:**
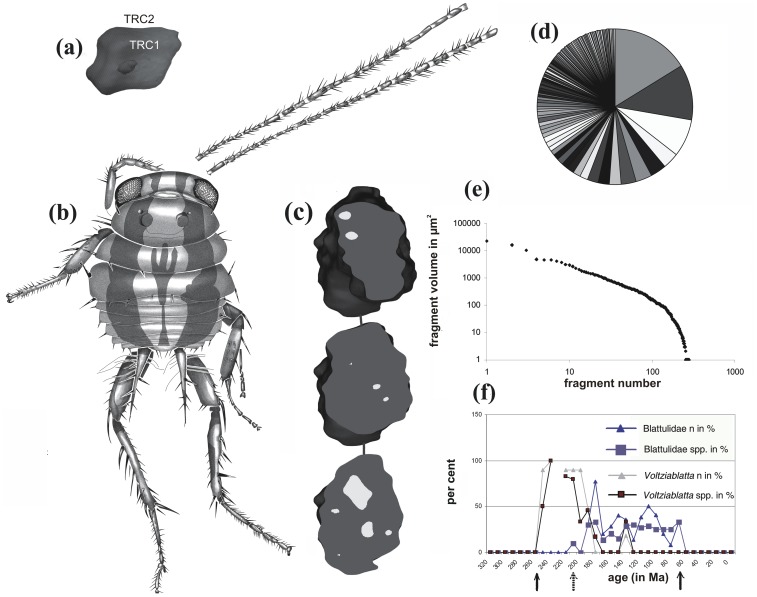
Dinosaur-age wood decomposing cockroach with coprolite and its ecological context. **A)** wood fragment no. 123 (coprolite no. 3), volume 23077 µm^3^ (TRC- parenchymatous tangential ray cells); **B)** Lebanese amber (Blattulidae 1094A-I), length (head to leg end): 3.8 mm; **C)** a virtual synchrotron section (∼1.2 mm) through coprolite no. 3, wood particles are pale; **D)** percentual representation of volume of the respective wood particles; **E)** distribution analysis of simple particle count of 280 wood fragments present in all five coprolites plotted over the fragment size; **F)** Ratios of the Blattulidae and “*Voltziablatta*”- group – families that replaced each other during the Triassic (interrupted arrow) – to all cockroaches, plotted over the timescale (in Ma). The origin and extinction of dinosaurs are pointed with arrows. “N in %” means percentual representation of number of specimens, “spp in %” is a percentual representation of species. Original data.

Distribution of the Blattulidae is associated with the abundance of dinosaurs (fig. 2F). In the Lebanese amber, the Blattulidae constitute 8 of the 15 identified (21 studied) cockroach samples including *Ocelloblattula ponomarenkoi* Anisyutkin et Gorochov, 2007 [36], in addition to the Umenocoleidae (n = 1), Caloblattinidae (n = 2), Raphidiomimidae (n = 1), Liberiblattinidae (n = 1), Blattellidae (n = 2), and Mesoblattinidae (n = 2; *Nymphoblatta azari*) [37].

The present fossil (Fig. 1) can be categorized as belonging to Blattulidae on the basis of small size, chaetotaxy and a significant comparative specimens of amber which include both immatures and adults [28], [38]. Its characteristics are a small size, large head, antennae with corrugated surfaces, and with 2–3 rows of long sensilla (Fig. 1BC), pronotum and abdomen with two longitudinal stripes, cerci with long spurs and extremely long sensilla, legs short. Especially notable are round elevated pronotal structures of the present nymph (see Fig. 2B), somewhat resembling lanterns (A lantern is a specialised light-producing organ of cockroaches.) of the luminescent cockroaches of the genus *Lucihormetica* [39], [40].

The diet of the Blattulidae is revealed for the first time. Five coprolites (the last one still protruding from the abdomen) that are elliptical in shape and circular in cross section (volumes 847,381 µm^3^, 2080,512 µm^3^, 2401,192 µm^3^, 3435,904 µm^3^, 4597807 µm^3^) (Fig. 1E, S1) amounting to a total volume of 13362,796 µm^3^, and about 0.35 mm long contain heterogeneous material. They are preserved in a single piece of amber, adjacent to a fossil of the Early Cretaceous cockroach, and represent a new type of trace fossil (coprolite adjacent to a preserved dead organism) that will be designated elsewhere. 1.06% (141,081 µm^3^) is filled by partially digested particles of wood. The structure of the wood is revealed on the largest particles and the lignin bilayer (part of the numerous parenchymatous tangential ray cells) is apparent on Fig. 2a and S1. The distance among parenchymatous tangential cells is roughly 10 µm.

The surfaces are smooth and the edges of the particles are rounded even in the largest particles (and also inside of cavities). The size of them (ca. 30,000 µm^3^) is still very small when compared to the mouthpart and mouthful size (e.g., particles of the cockroaches of this size often reach 0.4 mm at the widest point).

Wood within the present coprolites has a characteristic, possibly power law distribution of particles larger than 100 µm^3^ (distribution curve at Fig. 2F can be characterised with the equation y = −1.964x +10.695; y = log (size); x = log (number of debris)), but the frequency of smaller particles decreases (Fig. 2D) at 100 µm^3^, which is far enough to be recorded by the present technique (effective pixel sizes below 0.5 µm are common for the present synchrotron (ST)). The wood particles are not distributed concentrically and/or in an otherwise ordered way.

Additionally, this wood is apparently decayed in the hind gut (intestine and/or rectum - as in termites - not in mid gut or stomach) as the last incompletely formed coprolite (caused by stress-defecation and still extruding from the body) contains numerous larger wood particles (S1). This enhanced gut activity is documented by the amorphous structure of the coprolite apparent in the sections (Fig. 2C).

The distribution curve of the wood particles is ambiguous. The gut-processed particles are diminished below 100 µm^3^, which is the rough limit for the smooth edges caused by the cockroach gut-processing. On the other hand, the linear (in log scale) distribution of particles, combined with rounded edges in larger scale (up to 10,000 µm^3^) and the absence of small particles and isolated tracheae (only 3 linear particles are present, and they probably do not represent tracheae) in the present coprolite suggests external pre-digestion.

Dinosaurs apparently had consumed leaves along with the twigs, but the soft parts of leafs are unrecognizable in the ST signal. Only the hard and dense wood particles are distinct.

## Discussion

The most effective exploiters of nitrogen in animals are cockroaches, often capable of nitrogen extraction and symbiont transfer even from their own feces or from feces of vertebrates including the popular guano of diverse vertebrates. Its storage and transfer to conspecifics is thought to be used as currency in mating and parental investment strategies [12]. Cockroaches feed on the droppings of frugivorous, insectivorous, and haematophagous bats, but not carnivorous bats [41]. Insect communities on the dung of crocodiles, varanid lizards and big turtles are virtually unstudied, and bird dung is generally too small to be utilized by a specialized dung cohort [17]. Nevertheless, several living cockroaches are associated with bird nests and presumed to feed on bird dung [12], [42]–[46]. The only large volume bird dung of the oil bird *Steatornis caripensis* or guacharo (see Tab. 1) is processed by cockroaches [47], which is another (indirect) support for the present inferences as birds are direct descendants of dinosaurs (often systematically cathegorized directly inside them). Numerous authors [48] note explicitly but without specification direct utilisation of reptile dung. Christoffersen & De Assis [49]summarise pentastomid parasites transferred to cockroaches via feeding on reptile and amphibian feces (see Tab. 1). Although appearing trivial, cockroaches, one of the dominant insect orders during the Mesozoic were never examined as representing top candidates for partial processors of dinosaur dung.

**Table 1:** Distribuition of living dung-feeding cockroaches supporting their common and cosmopolitan distribution [41], exclusively in dark (nocturnal, cave or under dung) environments.

Species	Family	Locality	Country	Dung	Host	Habitat	Continent	Reference
*Arenivaga grata*	Corydiidae	Tucson Mountains,	USA, Arizona	guano	Bat	Bat cave	North America	[83]
*Blabverus discoidalis*	Blaberidae	Bogor, Java	Indonesia	feces	Flat-tailedgecko	Outdoors	Asia	[84]
*Blatta orientalis*	Blattidae	Johannesburg Hospital	South Africa	dung	Human	Hospital	Africa	[30]
*Blattella germanica*	Ectobiidae	?	Egypt	feces	Human	Villages	Africa	[85], [86]
*Ergaula* *scarabaeoides*	Corydiidae	Selangor	Malaysia	guano	Bat	Bat cave	Asia	[87], [88]
*Eublaberus distanti*	Blaberidae	Guanapo Cave	Trinidad and Tobago	dry guano	Fruit bat	Bat cave	South America	[4]
*Eublaberus posticus*	Blaberidae	Trinidad island	Trinidad and Tobago	feces	Bat	Indoors	South America	[89]
*Eublaberus posticus*	Blaberidae	Tamana cave	Trinidad and Tobago	guano	Oilbird	Bird cave	South America	[52]
*Euthyrrhapha nigra*	Corydiidae	Antsinomygrotto	Madagascar	guano	Bat	Bat cave	Africa	[90]
*Gyna kazungulana*	Blaberidae	?	East Africa	guano	Bat	Bat cave	Africa	[91]
*Gyna maculipennis*	Blaberidae	Lualaba	Dem Rep Congo	guano	Bat	Bat cave	Africa	[92]
*Opisthoplatia* *maculata*	Blaberidae	Formosa	Formosa( = Taiwan)	dung	Human	Outside	Asia	Shikano in [93]
*Paratemnopteryx* *kookabinnensis*	Ectobiidae	Kookabinna George	Western Australia	guano	Bat	Cave	Australia	[94]
*Paratemnopteryx* *rufa*	Ectobiidae	Nullarbor Plain	Australia	guano	Bird	Cave	Australia	[95]
*Paratemnopteryx* *weinsteini*	Ectobiidae	Rope LadderCave	Queensland	guano	Bat	Cave	Australia	[94]
*Parcoblatta bolliana*	Ectobiidae	Texas	USA	dry dung	Cow	Pine woods	North America	[96]
*Parcoblatta* *fulvescens*	Ectobiidae	Florida	USA	dry dung	Cow	Pine woods	North America	[97]
*Periplaneta* *australasiae*	Blattidae	Sarawak Mt. Jibong	Malaysia	guano	Bird	Cave	Asia	[98]
*Periplaneta* *australasiae*	Blattidae		Malaysia	feces	Small reptiles	Outdoors	Asia	[99]
*Periplaneta* *australasiae*	Blattidae	Punta Gorda, Florida	South Africa	dung	Goat	Outside; vacanthouse	North America	[100]
*Periplaneta* *americana*	Blattidae	Formosa	Formosa( = Taiwan)	feces	*Macaca* *cyclopis*	Indoors	Asia	[101]
*Periplaneta* *americana*	Blattidae	Vengurla	India	guano	Bat	Bat cave	Asia	[102]
*Periplaneta* *americana*	Blattidae	Sumatra Sawah Lunto	Indonesia	feces	Human	Coal mine	Asia	[103]
*Periplaneta* *americana*	Blattidae	western Bengal	India	feces	Human	Coal mine	Asia	[104], [105]
*Periplaneta* *americana*	Blattidae	Johannesburg Hospital	South Africa	dung	Human	Hospital	Africa	[30]
*Periplaneta* *americana*	Blattidae	?	Egypt	feces	Human	Villages	Africa	[85], [86]
*Periplaneta* *americana*	Blattidae	Accra –laboratory	Ghana (GoldCoast)	feces	*Erythrocebus* *patas*	Indoor (glassjars)	Africa	[106]
*Periplaneta* *americana*	Blattidae	Araripe	Brazil	feces	Worm lizard	Outdoors	South America	[107]
*Perisphaerus* sp.	Blaberidae	Jalor caves	Malaysia	guano	Bat	Cave	Asia	[108]
*Pycnoscelus* *surinamensis*	Blaberidae	St. Croix	USA, VirginIslands	feces	Chicken	Chicken roosts	CentralAmerica	[109]
*Pycnoscelus* *surinamensis*	Blaberidae	Puerto RicoMona Island	USA	dry dung	Cow	Pine woods	CentralAmerica	[43]
*Pycnoscelus* *striatus*	Blaberidae	Selangor	Malaysia	guano	Bat	Cave	Asia	[87], [88]
*Simandoa* *conserfariam*	Blaberidae	SimandouMts.	Guinea	guano	Fruit bat	Cave	Africa	[110]
*Symploce cavernicola*	Ectobiidae	Sarawak Mt.Jibong	Malaysia	guano	Bird	Cave	Asia	[98]
*Tivia* *macracantha*	Corydiidae	Katanga Province	Dem Rep Congo	guano	?	Cave	Africa	[92]
*Tivia* sp.	Corydiidae	Antsinomygrotto	Madagascar	guano	?	Cave	Africa	[90]
*Trogloblattella* *nullarborensis*	Ectobiidae	NullarborPlain	Australia	guano	Bird	Cave	Australia	[95]
*Xestoblatta* *hamata*	Ectobiidae	La Selva	Costa Rica	dung	Bird	?	Cental America	[4]
*Xestoblatta* *immaculata*	Ectobiidae	Chilibrillo	Panama	guano	Bat	Cave	Cental America	[111]
*unidentified*	?	?	?	dung	Horse, Cow	Desert	?	[112]
*unidentified*	Corydiidae	?	Ecuador	dung	Bird	Outdoors	South America	[12]
*unidentified*	?	?	Malaysia	feces	House gecko	Indoors	Asia	[113]
*unidentified*	?	Hawai	USA	feces	Giant toad	Outdoors	North America	[114]

Feeding of diverse cockroaches on bird excrements and also facultative feeding on reptile and amphibian dungs is apparent. Based on Bell et al. [12], Christoffersen & De Assis [49] and Roth & Willis [115].

The present specimen represents a derived secondary trace within a trace (traces of microorganisms on wood preserved in a coprolite–a trace of a cockroach within amber–a trace of a tree). Although it represents a unique find in respect to both quality of preservation in amber as well as the incidental character of the preserved “act”, coprolite feedings of Mesozoic cockroaches from other families can be excluded based on the positive evidence in the form of preserved gut contents. Several dozen species from the sedimentary record of diverse families (Mesoblattinidae, Caloblattinidae, Ectobiidae, Liberiblattindiae, Umenocoleidae) were found with the gut content. All of them contain unprocessed heterogenous organic debris, but no wood (unpublished observation), which is irreconcilable with coprophagy. Thus the only family adept for such duties is the family Blattulidae–the last ecologically significant family with unstudied gut content. The generic diversity of this family was significantly low, namely only 12 genera are present in their 80 million years of ecological dominance. This low diversity is also represented in the fossil inventory of the Lagerstätten and is direct evidence for very uniform, constant niches and probably also for a more or less uniform diet. This phenomenon is also visible in the unusually minor differences between genera of the sedimentary and amber records. This minimal diversity is highlighted to a greater extent by the sparse disparity. With the exception of two rare species, all Blattulidae are very similar. Uniformity is especially shown by the transversally striated extremities. This coloration dominates in the whole Mesozoic, but was lost at the K/Pg boundary along with the extinction of dinosaurs, although this colouration occurs in extant, nocturnal and arboreal *Allacta australiensis* under different body colors.

Just a lack of diversity could mean it had a limited niche, one that could be seen in modern roaches, but combined with the longest lasting ecological dominance within cockroaches and unique morphology (such as corrugated surface of antennae–Fig. 2B,C), indicating the niche of the Blattulidae was different from that of living cockroaches.

Generally, during the Mesozoic representatives of the family Blattulidae usually comprise ∼1% of all insects and over 30% of cockroaches (Fig. 2F), and thus were probably associated with a dominant group of vertebrates–probably sauropod dinosaurs. Special features of the present specimen such as extremely short and wide body with very long cerci suggest it is closely related to *Grandocularis kurnubinsi* from Jordanian amber (described based on a nymph [50] of a similar stage and size). It apparently represents a closely related species, but differs in the form of the pronotum, eye size, coloration and chaetotaxy. In adults, bioluminescent “lanterns” were apparently absent–adults of at least several species of the Blattulidae were documented as crepuscular or diurnal, not nocturnal–on the basis of the eye morphology and common occurrence together with diurnal species within a single pterosaur and/or dinosaur coprolites and/or regurgites [51]. Cockroach nymphs occurring in dung would signal to adult ovipositing females by a lantern system. But the detection of luminescence of lanterns embedded in amber would be difficult. Unfortunately, the ST signal in a large piece of amber is too weak even to reveal morphological details and thus the presence of these morphofunctional units cannot be validated.

One can imagine the distinct contrast coloration characterized by distinct alternating light-and-dark stripes would be advantageous (for communication) in an open and confined habitat of dung surfaces. On the other hand, neither cockroach guano dwellers nor recent “external” coprophages have any conspicuous coloration. Additionally, all living coprophagous cockroaches live concealed within and/or under dung. In nocturnal conditions of caves, nymphs also burrow in the surface of loose guano. They may be completely concealed, or may rest with their heads on the surface with their antennae extended up into the air; if the guano is compacted, the cockroaches remain on its surface and are attracted to irregularities such as the edge of a wall, a rock, or even a footprint [52]. In these dark conditions, guano cockroaches are also present on dung and mostly are absent from cave zones of dry soil, stones, or pebbles [53], [54].

The low diversity may be a consequence of a heterogenous diet and/or low specialization of herbivorous animals of which dinosaurs were the most abundant (suggesting there was relatively little nutritional variability in their excrement and thus less need for specialized roaches). Low specialization of at least some dinosaurs is confirmed by phytoliths extracted from the Upper Cretaceous coprolites (from dicotyledons, conifers, and palms) from India, suggesting that the suspected dung producers (titanosaur sauropods) fed indiscriminately on a wide range of plants, including grasses [7]. With the diversification of mammals [55], diverse specialized dung-beetles co-evolved [2] and these cockroaches, possible with low specialization in their feeding behaviors became extinct.

Generally, before the massive radiation of the Blattulidae at the beginning of the Jurassic, their niche was occupied by the superficially similar “*Voltziablatta*” group of cockroaches, which became extremely rare along with the radiation of the Blattulidae. In all Mesozoic sites, “*Voltziablatta*” and the Blattulidae occur in congeneric species pairs, discretely differing in size, but not in general appearance, thus doubtfully representing nocturnal and diurnal cohorts (occurrence of both sexes in both groups was validated earlier [51]). This enigmatic observation is unexplained and needs further investigation. The *Voltziablatta* group phylogenetically connects its descendants, the herein studied Blattulidae and living cockroaches which bear endosymbionts; namely termites, *Sociala* and *Cryptocercus* all descended from Liberiblattinidae. If this mutualism had a single origin, it must have been in the *Voltziablatta* group (fixed to flora and wood of *Voltzia* plants), where the lignin consumption must have originally evolved. In the opposite case, we would need to consider three independent origins of endosymbionts, which molecular data do not support [56].

### Coprolite and Dung Decomposition

Presence of related endosymbionts in termites and cockroaches of the family Cryptocercidae was postulated to be an evidence for their direct relation. Nevertheless, the probable presence of endosymbionts in the Mesozoic clade which diverged from stem of higher cockroaches explains the monophyletic origin of these symbionts in both groups also in the phylogenetic reconstructions where they are not directly related [3]. The question is why was this capability lost in most regular cockroaches?

The hypothesis that lignin-decomposing insect and their endosymbionts originated via the consumption of wood pre-digested by herbivore animal needs explanation. Feeding on lignified wood and also foliage-eating became more widespread in both dinosaurs and insects only with the radiation of angiosperms at the Early Cretaceous/Late Cretaceous boundary [1]. Dung consumption by Mesozoic termites, assisting in decomposition of processed plant matter was already proposed [14].

Even the wood decay is preserved in a single sample, it is clear that these cockroaches might have employed at least a semisocial way of life to provide the horizontal endosymbiont transfer (thus supporting the view that it evolved just once, as confirmed by the phylogenetical scheme). In recent tropics, where food is available for bats throughout the year, guano deposition is predictable and also supports very large, persistent groups of cockroaches–guanobies [57].

To summarize the arguments supporting dung processing, this single sample is decisive in showing a coprolite still extruding from the body (and thus belonging to the body fossil as a producer, excluding incidental preservation) and containing modified wood fibres with typical parenchymatous tangential ray cells. Lignin can not be processed this way without endosymbionts and even in the case it has been modified to some extent by some fungi, it must have been pre-processed externally. The wood was apparently processed before it entered the cockroach digestive tract as indicated by the large extent of digestion apparent in cavities (which definitely exclude the mechanical processing) and the fragment preservation plotting fragment volume over the fragment number–Fig. 2e; additional indirect support comes from dung-processing of living cockroaches, Tab. 1. It must be stressed, that the extent of smoothing of large particles including large cavities excludes the exclusively within insect processing and is evidence for external pre-digestion. In this respect, a source of the wood directly from the environment can be excluded. There are only three possibilities for the pre-digestion, namely the fungal (excluded below based on selective disadvantage of preference of large indigestible particles and absence of wood damages before the Late Cretaceous contrasting with plethora of coprolites containing wood) and vertebrate pre-processing or their combination. Large particles are numerous indicating that they were not selectively avoided during consumption. Underrepresentation of smaller particles was apparently due to biochemical digestion of wood lignin as do their eusocial (extinct cockroaches of the family Socialidae and termites) and semisocial (Cryptcercidae) descendants. Although it is very probable that dinosaurs preferred wood processed by fungi, fungi-only pre-digestion and feeding of these cockroaches can be excluded based on the presence of large fragments combined with low partition of wood. Such a small amount would suggest selective feeding on fungi-modified wood, in which circumstances large particles are contradictive; on the contrary, unselective feeding on coprolites would contain the expected spectrum of particles of diverse size. The only possible explanation is that these were caused by herbivorous vertebrates. Due to the dominance of these cockroaches for the same 200 million years as dinosaurs, no other vertebrate group is as promising for this candidature. It can not be excluded that cockroaches also cleaned up after some small, unknown vertebrate herbivores, but these can be excluded from the present study as small vertebrates can not digest wood.

Certainly, in such a case, in any solitary taxa the capability of symbiont transfer and thus utilizing lignin was necessarily lost. Termites did not exist before the Middle Jurassic, but their precursors under study were apparently pre-adapted for wood decomposition – and thus possessed one of the necessary conditions for the origin of a eusocial way of life. Nevertheless, termites were diversified in the very beginning of the Cretaceous as evidence from the presently studied locality in Lebanon also indicates [3], [58], [59].

Transfer of microflora within dinosaurs was proposed via juvenile coprophagy [60], which facilitates microflora but also endoparasite transfer with cockroaches. It is actually the intestinal bacteria and metabolic by-products [61], [62] of the herbivore gut (perhaps dinosaurs), which likely allowed for lignin digestion in Blattulidae (by protozoans). The small proportion of wood content (∼1% is of only partially processed wood remnants and up to 5% of completely processed wood, not recognized in the ST) in the cockroach coprolite indicates that wood was not the primary constituent of the diet of the present individual, and rather supports the derived source. This is also indicated by the Late Cretaceous dung of herbivorous reptiles [63], probably dinosaurs (entirely of comminuted plant tissue with the predominance of secondary conifer xylem tissues of Cupressaceae). The unmodified state of the cells and the absence of gymnospermous wood in dung [64] is still problematic, but the small size of the plant fragments infilling the fossil burrows suggests comminution or sorting by invertebrates [63]. Also several gymnosperms remains (Cheirolepidiacae and Araucariacae) were found in the unstudied coprolite (larger than the present ones) from the same deposit in Lebanese amber.

The distance among parenchymatous tangential cells of the wood in the present coprolite is roughly 10 µm, which is comparable to the structure of wood of fossil *Taxodioxylon vanderburghii* or *Metasequoia glyptostroboides* (20–30 µm [65]). Even more similar parenchymatous tangential cells (10–20 µm) are found in unidentified conifer wood from dinosaur coprolites (as indicated in Fig. 5B, upper part of [8]). Interestingly, this wood originates from trees growing in warm and semiarid Late Cretaceous environments preserved in the sediments of the Two Medicine Formation [8], which is in contrast to the warm and humid amber-producing Early Cretaceous forest of Lebanon. Anyway the specific determination of fossil conifer woods is very difficult and requires comparisons of many features that do not seem to be present in the small particles of wood in the fecal pellets.

The wood (the length of the largest fragment was 13 cm) preserved in dinosaur coprolites is characterized by absence of cylindrical wood stems (no terminal twigs were digested); damage to lignin such as the presence of pliant tracheids, uneven cell walls and deformed and missing cells is also characteristic [8]. This, along with the fact that the vertebrate gut cannot hold complex lignolytic organisms, because these protists are anaerobic suggests fungal decay prior to consumption [8]. On the contrary, the small amount of small wood particles in these coprolites indicates they were processed within dinosaurs and support decomposition of the smallest particles both in dinosaur and cockroach coprolites.

### Coprolite and Dung Decomposition-defecation

In spite of the diversity of behaviors reported from amber, a review by Arillo [66] contains a single defecation, reported from a Dominican amber termite [67]. Nevertheless, there is a rich Cretaceous termite record of distinctive fecal pellets with diagnostic hexagonal cross-sections that commence during the Hauterivian or Barremian [68] and continue to occur in various woods to the end of the Cretaceous. Some of these pellets may have originated from individuals belonging to taxa such as the eusocial cockroach *Sociala* that occurs in Mesozoic amber [3]. Fecal pellets from wood are known [69], and most amber coprolites contain wood remains and are assigned to wood borers among termites, beetles or some other insects [70], [71]. Additional pellets are known from the Dominican amber [72] and frass containing fungi are known from Archingeay amber [73]. Defecation was probably often associated with escaping behaviour, because more than 60 samples of Lebanese amber (coprolites are often separated) contain coprolite of diverse size and shape (large elongate, oval). Lots of them were preserved with wood fibers. In the same piece there are insects like ceratopogonids, chironomids, archizelmerids (extinct flies) and wasps, but these coprolites are not associated with insects and are mentioned here to demonstrate the common defecation behaviour, not the wood processing. No trace fossils documenting specialized dung provisioning are known before the Late Cretaceous [2].

### General Ecology of Dung Provisioning

Detritic food chains strongly predominated in the Mesozoic [2] and the dominance of the Blattulidae among cockroaches seems to be associated with dung being the most valuable source of nitrogen. It is improbable that there were specialized guilds of dung feeders in the Mesozoic comparable with modern regarding structural complexity and ecological efficiency: Sciaridae and Scatopsidae (flies) with rapid larval development were remarkably rare [74], as well as dung beetles, although both are present in the Lebanese amber [75] along with decomposer flies of the families Psychodidae and Sciaridae. However, they were absent before the Jurassic and extremely rare during the entire Jurassic [10], [64], [75].

Alternative opportunistic exploiters of dinosaur dung were snails. Multiple associations of 132 (with 0–66 specimens each) fossils (*Megomphix, Polygyrella*, *Hendersonia, Prograngerella,* and three aquatic taxa) have also been observed on or within 6 of the 15 herbivorous dinosaur coprolite deposits [15].

Despite the great diversity and quantity of scarabeid beetles in the Mesozoic ([10] especially in the Middle Jurassic locality Daohugou in Inner Mongolia, China), only a few species can be considered as possibly coprophagous. Only 3 dung ball-makers from the subfamily Scarabaeinae are known: *Prionocephale deplanate* (Upper Cretaceous Lanxi formation, Zhejiang, China [76]), *Cretonitis copripes* (Early Cretaceous Zaza Formation, Baissa, Russia) and an undescribed species [72], [77] of the living coprophagous genus *Trypocopris*. Representatives of the Geotrupidae were probably coprophagous: *Parageotrupes incanus* from the Yixian Formation [78], and *Cretogeotrupes convexus* and *Aphodius* (s.l.) (Aphodiinae) from Baissa [77], [79].

An alternative hypothesis claiming mainly aquatic plant diet of dinosaurs [80] and thus water defecation does not explain at least some damage to terrestrial plants.

The dung of known Mesozoic herbivores is composed mainly of undigested fern and gymnosperm tissues and was utilized by opportunistic detritivores together with other plant litter [2].

The specialized coprophagy by beetles is recorded as late as the Late Cretaceous when the diet of grazing dinosaurs apparently contained angiosperms other than grasses and ecosystems were based on biomes similar to grasslands [1]. Based on our investigations, pollen and angiosperms in the Lebanon amber are indicated by at least 5–6 different taxa.

The decay of wood pre-digested in dinosaur gastrointestinal tracts explains and predicts the single origin of lignin consumption in the common ancestor of termites, eusocial cockroaches (*Sociala*), and semisocial cockroaches of the family Cryptocercidae. It would also explain a huge number of termite-like fecal pellets (containing wood) in Mesozoic ambers with parallel absence of any termite damage to wood [68]. The fact that termites were a major lineage responsible for the degradation of plant tissues (when compared with cockroaches) is irrelevant in this respect as they originated not earlier than in the Middle Jurassic when their ancestors, certain Liberiblattinidae appear in the fossil record [4] and thus can not play any role in the decomposition of early sauropod dung. In contrast, blattulid cockroaches and their ecological equivalents originated as early as the Permian–Triassic boundary.

The contemporary robust appearance of Cryptocercidae does not require a major morphological shift from anticipated dung-beetle-habits. It is likely that dung processors will also lose wings like Cryptocercidae, but in caves, wing loss and associated morphological changes occur more frequently in organisms that rely on plant debris than those that rely on guano [81]. Under all circumstances it is apparent, that termite and cryptocercid ancestors were pre-adapted for lignin decay and, likely, provided a limited sanitation to herbivorous reptiles. Based on the correlation of distribution of reptiles and the dominance of the blattulid cockroaches in Mesozoic ecosystems, and their coeval occurrence in the present amber-bearing strata [82], these herbivorous reptiles were most likely the dominant sauropod dinosaurs.

## Supporting Information

Figure S1 **Synchrotron imaging of 5 coprolites of dinosaur-age immature cockroach from the Lebanese amber (Blattulidae 1094A-I).** Select transparent mode for 3D visualization and rotation.
(PDF)
