# Supplementary figures and images for: Cockroaches Probably Cleaned Up after Dinosaurs
Source: PLoS One. 2013 Dec 4;8(12):e80560. doi: 10.1371/journal.pone.0080560 (PMC3851186; doi:10.1371/journal.pone.0080560)

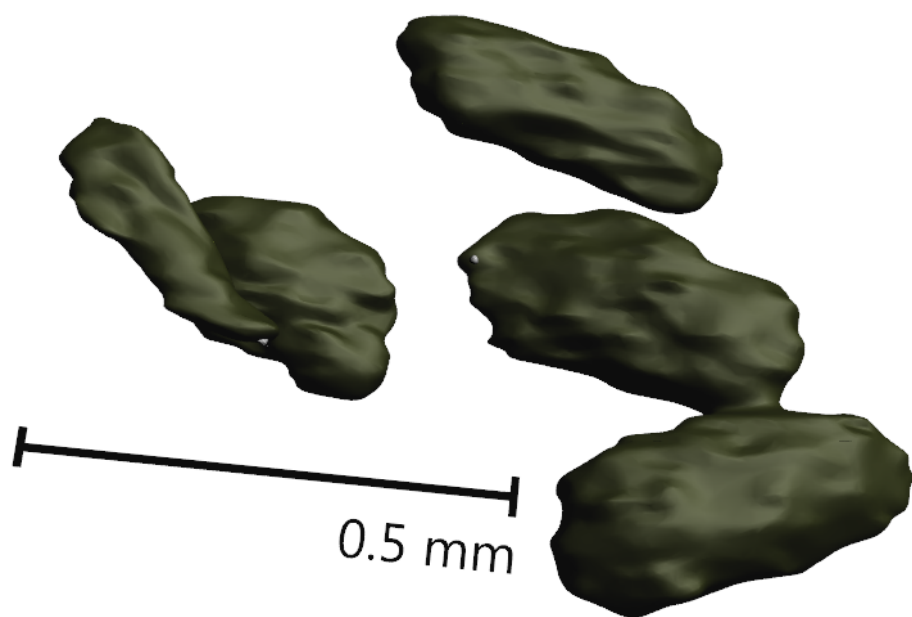

Supplement: Figure S1 — Synchrotron imaging of 5 coprolites of dinosaur-age immature cockroach from the Lebanese amber (Blattulidae 1094A-I). Select transparent mode for 3D visualization and rotation. (PDF) [file pone.0080560.s001.pdf]
